# Avian Bornaviral Ganglioneuritis: Current Debates and Unanswered Questions

**DOI:** 10.1155/2020/6563723

**Published:** 2020-01-19

**Authors:** Su L. Boatright-Horowitz

**Affiliations:** Psychology Department, University of Rhode Island, Kingstown, RI 02881, USA

## Abstract

Avian bornaviral ganglioneuritis, often referred to as parrot wasting disease, is associated with a newly discovered avian virus from the taxonomic family Bornaviridae. Research regarding the pathogenesis and treatment for this disease is ongoing, with implications for understanding other emerging human and nonhuman diseases, as well as the health and ecology of wildlife. At this time, numerous questions remain unanswered regarding the transmission of the disease, best practices for diagnostic sampling and testing, and whether currently used drug therapies are effective or harmful for afflicted birds. The pathogenesis of the disease also remains unclear with many birds showing resistance to the effects of the virus and being able to remain clinically unaffected for years, while other birds succumb to its effects. New research findings regarding avian bornaviral ganglioneuritis are discussed and important as yet unanswered questions are identified.

## 1. Introduction

Recently, there have been major advances in our understanding of avian bornaviral ganglioneuritis (ABG), sometimes called parrot wasting disease, including research about its causes, transmission, diagnostic testing, and treatment with implications for human medicine and the veterinary care of other animal species. It was previously referred to as proventricular dilatation disease (PDD) because symptoms can involve gastrointestinal crisis; the disease is now understood to have more extensive nervous system involvement, so it is more often referred to as avian bornaviral ganglioneuritis (ABG). There is also new evidence of a similar condition referred to as avian ganglioneuritis (AG), depending on whether the animal tests positive for the bornaviruses. Advances in our knowledge about the treatment and prevention of viral diseases such as the avian bornaviruses have important implications for veterinary practices, as well as the global ecology and the potential spread of zoonotic diseases.

## 2. Symptoms of the Disease

Clinical symptoms vary in both severity and type across individual birds, but they appear to be neurological in origin with clinical signs related to their effects on the digestive system and the nervous system. Gastrointestinal (GI) signs may involve excessive regurgitation, poor appetite, crop impaction, weight loss, and passage of undigested food in the feces. These symptoms may be related to pathology of the vagus nerve that controls the upper part of the digestive tract, including the crop, proventriculus, and ventriculus, resulting in reduced gastrointestinal motility [1]. Some researchers have also suggested that the interstitial cells of Cajal that control muscular movements in the digestive system are likely to be involved as the target of avian bornaviruses [2]. Regardless, with progression of the disease and high susceptibility, there can be paralysis of the intestines with food becoming stuck in the bird's proventriculus, a rod-shaped organ in the bird's digestive system located between the crop and the gizzard. With dysfunction of the vagus nerve or other digestive processes, this portion of the intestines can swell with blocked food and then rupture, resulting in a bird's painful death. Therefore, bornavirus infection should be suspected if there are weight loss, undigested food in the bird's droppings, vomiting, abdominal extension, and moaning with physical discomfort [3]. Bornaviruses can spread through other parts of a bird's nervous system and cause shaking of the head, abnormal and uncoordinated movements, difficulty balancing, tremors, paralysis, self-mutilation, aggression, and seizures. There may also be heart arrhythmias, blindness, and cognitive deficits, depending on the various locations of neurological damage [1, 4]. A survey of 32 bornavirus-positive birds from Brazilian clinics and breeding facilities, including several confiscated from illegal trade, revealed that 66% of afflicted birds showed CNS symptoms, while 22% had GI signs, and 9% of the birds died [5]. Some new evidence also suggests that feather-plucking is associated with this disease. In a study of 126 birds in a private veterinary practice in Germany, antibody titers and viral shedding were highest for birds with neurological signs of the disease, second highest for feather-plucking birds with no other neurological signs, and lowest for a control group without neurological signs or feather-plucking [6].

## 3. What Are the Bornaviruses?

It is commonly accepted today that parrot wasting disease or avian bornaviral ganglioneuritis can be caused by avian bornavirus infection. In 2008, parrot wasting disease was shown to be associated with a newly discovered virus, i.e., the avian bornaviruses which shared only 70% of their nucleotide sequence with the previously identified mammalian bornaviruses [7, 8]. Bornaviruses, members of the taxonomic family Bornaviridae, were named after the town called Borna in eastern Germany which had historically high occurrences of neurological disease outbreaks in horses and sheep. An epidemic among cavalry horses occurred between 1894 and 1896 with symptoms such as head-tilting, paralysis, aggression, and difficulties with chewing and swallowing. Research has shown that the virus occurs at a higher than normal frequency in both horses and sheep in this region, although bornaviruses occur in many other species of birds and mammals and in many other parts of the world, although it is difficult to assess the prevalence of the virus across species/geographic regions because blood testing procedures are not standardized, i.e., sample collections of serum vs. whole blood, testing for the presence of the virus itself or antibodies to the virus [9]. Bornaviruses consist of enveloped nonsegmented viruses with negative-stranded RNA genomes of approximately 8,900 bases (NNN). Replication and transcription of these viruses take place in the nuclei of cells, specifically nerve cells. As RNA viruses, they lack the enzymatic corrective mechanisms in replication that characterize DNA viruses. Without corrective mechanisms, RNA viruses are more likely to have changes in their genetic makeup so that the production of effective vaccines may become more difficult [10]. The impact of bornavirus infection appears to depend on the body's immunological responses because the presence of CD3+ host T cells in the brain has been shown to be strongly associated with the onset of neurological symptoms [11]. Furthermore, research has shown that the amount of virus detected in tissues after death is not associated with severity of clinical symptoms, supporting the conclusion that the body's immune response is a causal factor in the disease, although the specific pathogenic mechanisms are currently under debate by researchers [1, 2].

## 4. Transmission of Avian Bornaviruses

The transmission of bornaviruses from one bird to another is not fully understood. It makes sense to expect the disease to be transmitted horizontally through direct contact or through ingestion of an infected bird's urofecal matter. Research has demonstrated that aviaries with infected birds may have positive air tests for bornaviruses, presumably as a result of feather dust; therefore, it has been suggested that the virus can become aerosolized when infected birds defecate or regurgitate and then spread as these substances become dry [3]. On the contrary, investigations of transmission of bornaviruses in laboratory settings have resulted in inconsistent contagion results for noninfected birds housed in the same aviaries as infected birds [12]. Some researchers believe that horizontal transmission requires a long-term and close contact between birds [13]. Furthermore, unlike research with the mammalian bornaviruses [14], attempts to inoculate uninfected birds with bornaviruses via mucosal surfaces (nasally and orally) have thus far had inconsistent results [15]. Instead, successful experimental inoculations of birds have included intramuscular injection [16], as well as intracerebral and intravenous injections of the virus [17]. It remains unclear why there would be such a major difference between birds and mammals in the effectiveness of exposure to the virus through nasal and ocular entry sites [18] or why these routes would be effective while the virus is believed to be spread through axonal transport processes [16]. There is remarkably little speculation about this in the published literature. Perhaps, it is related to local anatomical or physiological differences between these classes of animals, discussed for chickens vs. mammals [19], or the stability and viability of the avian bornaviruses in the biological systems of birds [20]. On the contrary, it may involve differences in innate immune system responses, for example, the lack of an anti-inflammatory protein identified in chickens [21]. Regardless, these are important questions regarding the transmission of avian bornaviruses and the immune systems of birds that remain unanswered.

Given the effectiveness of intramuscular and intravenous injections for bornavirus infection, it may also be speculated that cutaneous wounds provide opportunities for infection. Recent reports of three human deaths due to encephalitis following bites or scratches by variegated squirrels positive for the mammalian bornaviruses would seem to support this possibility [22]. Researchers have also examined vertical transmission of bornaviruses by testing the contents of eggs laid by infected birds. While the results were positive, this research has not yet included systematic procedures to allow the eggs to hatch in order to determine the effects of bornaviruses on parrot hatchlings [23]. Exposure to avian bornaviruses in unweaned chicks tends to result in a rapidly high mortality rate compared to older birds, perhaps due to an undeveloped immune response. To date, one research study has examined vertical transmission of bornaviruses from parents to hatched offspring in free-ranging birds (i.e., Canada geese) showing negative results [23]. In sum, although bornaviruses have been observed to have high contagion in some aviaries, laboratory testing of the spread of the virus still has not identified a specific reliable route of infection, although some researchers now suggest that vertical transmission is likely to be shown to be the primary cause of avian bornavirus transmission [3], despite yet unanswered questions regarding mortality rates in infected hatchlings and the current lack of information about the timing of viral transmission during egg development.

## 5. Diversity in Subtypes of Avian Bornaviruses

In the 1970s, when scientists became aware of the occurrence of parrot wasting disease, there were few, if any, regulations regarding the capture of parrots in their native habitats for the pet market in the US. These animals were often housed in large quarantine warehouses that lacked protocols to prevent the spread of diseases. Many birds suffered and died as a result of these conditions. Without a doubt, there was enormous stress for these animals associated with being removed from their families and homes, only to be housed in cages, often in unhygienic conditions with uncontrolled mixing of different bird species. Interestingly, genetic analysis does not support the view that parrot bornaviruses were transferred from waterfowl during this time [24]. Yet, these stressors likely enhanced vulnerability to the disease and contributed to the high mortality rates for these birds, perhaps impacting the subtypes of bornaviruses identified in captive parrot populations today. At the last count, researchers mapping the genomes for bornaviruses in birds have identified a total of fifteen genotypes of bornaviruses, 12 classified and 3 unclassified within the genus *Orthobornavirus* [25], which can occur in avian species, including eight found in parrot populations worldwide [15] that share 91 to 100% of their nucleotide sequences within genotypes and 68 to 85% between genotypes [26]. It has been speculated that the diversity of bornavirus genotypes in captive parrots compared to free-living waterfowl is the result of the spread of bornaviruses across species during the unregulated housing and transport associated with wild captures decades ago [6]. Genotypes for bornaviruses in parrots do not appear to be clearly linked to geographical regions unlike bornaviral genotypes identified in waterfowl [15]. The genotypes identified as most virulent for parrots are subtype numbers 2 and 4 (referred to as PABV-2 and PABV-4), and these are the usual targets of bornavirus testing for veterinary purposes today [15]. Note that if bornavirus tests are negative and veterinarians still suspect the disease, then it may be necessary to expand testing to other subtypes of the virus [3]. Some research findings with cockatiels revealed differences in the effects of these two strains of the virus with PABV-4 associated with more neurological signs, while PABV-2 mainly affected the gastrointestinal tract with more severe disease progression, although other factors may play a role, including individual immune responses and how well a virus is adapted to a particular host species [5]. Simultaneous presence of both genotypes appears to be associated with more severe clinical signs with infection with one subtype failing to provide protection against the other [13]. On the contrary, tracking the spread of PABV-2 in individual cockatiels following intramuscular injections revealed the presence of the virus at the inoculation site and adjacent nerves, then in the spinal cord, and finally in the brain. With CNS infection, the virus also spread to the gastrointestinal system, adrenal gland, heart, and kidneys over 114 days of experimentation [16]. Clearly, additional larger scale studies with multiple species are required to further investigate the relationships between bornavirus subtypes, clinical symptoms, and histopathology in captive parrots. It is also well known that many birds who test positive for bornaviruses, including PABV-2 and PABV-4, do not show symptoms. In fact, recent estimates indicate that 10% to 45% of captive birds are infected, and at least one in three healthy captive parrots is likely to test positive for bornaviruses. Furthermore, some free-ranging Brazilian parrots in their natural habitats have been shown to be positive for bornaviruses [27]. However, others pointed out that these birds had been housed in rehabilitation centers for weeks, allowing for infection during this time period [15]. Clearly, more field studies are needed with long-term tracking of infected birds combined with genetic studies of viral subtypes.

## 6. Is It an Immune or Autoimmune Response?

Another important debate about avian ganglioneuritis involves the pathogenic mechanisms for this disease. It is generally believed that avian bornaviruses affect the body's immunological response because CD8+ host T cells in the brain are strongly associated with the onset of neurological symptoms and the amount of virus detected in tissues after death is not associated with severity of clinical symptoms [1]. Specifically, CD8+ T cells cause direct damage to the ganglia and neurons, combined with CD4 T-cell recruitment of macrophages, ultimately resulting in antibody-mediated phagocytosis of axons [1]. Less direct mechanisms including loss of protective myelin, dysfunction of mitochondria, and release of nitric oxide or glutamate may contribute to axonal damage [1]. Both innate and adaptive immune responses appear to be involved because complement components C1 and C3 tend to be present inside avian bornaviral lesions, suggesting that both the classic and alternative complement cascade pathways are activated, respectively [1]. However, the alternative or innate pathway can be triggered as a function of foreign substances and damaged tissues, thereby amplifying the activation cascade. Some researchers have suggested that an autoimmune response may be involved as host antibodies attack gangliosides located in neural cell membranes. In 1942, Ernst Klenk first used the term “ganglioside” to describe lipids isolated from ganglion cells in the brain. Today, we know that gangliosides are primarily located in the nervous system and that they are microscopic structures that protrude through the surface of the cell to act as surface markers for cellular recognition and cell-to-cell communication [28]. Rossi and his colleagues suggested that an autoimmune mechanism may occur with inflammation of the nerve ganglia causing exposure of normally protected proteins (i.e., gangliosides) to the host immune system in a fashion comparable to Guillain–Barré syndrome in humans [1]. Recently, researchers in the Netherlands published a paper outlining the role of these cell structures as modulators of immune functioning [29]. However, de Araujo et al. argued that Guillain–Barré syndrome is associated with peripheral neuropathy and limb weakness which are not primary features of proventricular dilatation disease [30]. They suggested that ambulatory dysfunction in afflicted birds, if it occurs, is more likely due to CNS lesions. They further pointed out that macrophages play an essential role in the development of nerve lesions in Guillain–Barré syndrome, specifically causing demyelination which is not characteristic of proventricular dilatation disease in parrots. In order to test for autoimmunity as a causal factor for the disease, these researchers inoculated healthy chickens and Quaker parrots with brain gangliosides or crude nervous system extracts via the pectoral muscles and reported that it did not result in clinical signs of the disease after 114 days [30]. However, in a criticism of this work, Rossi et al. indicated that some of these birds did exhibit some pathological signs of the disease, but they were discounted by the researchers as naturally occurring lesions in the gastrointestinal tract or CNS of avian species which occur with inoculations of foreign substances [1]. Rossi and his colleagues further pointed out that one chicken was reported to develop a mild difficulty walking and two of five parrots showed mild depression, while a third parrot evidenced weight loss, and one parrot was observed to have depressive symptoms [1]. Notably, the parrot with weight loss had the highest titers for antiganglioside antibodies tested with ELISA [1]. In contrast, Rossi and his colleagues described their experiments in which young cockatiels were inoculated with gangliosides extracted from the CNS of uninfected parrots and reported that it resulted in clinical signs of the disease and typical histological lesions, as well as high ganglioside antibody titers [1]. This suggests that the disease can be manifested in the absence of bornaviruses when there is comparable damage to the nervous system. As these researchers indicated, it is not yet clear how antiganglioside antibodies contribute to the progression of disease, but investigations of autoimmune involvement may allow for a clearer understanding of the complex pathogenesis of this disease, and one might speculate that it could help explain the high levels of individual variability among parrots in susceptibility to the disease [1]. Other researchers have concluded that some individual parrots may exhibit transient autoimmune responses to the disease, stating that they are not a common response [31].

## 7. Diagnostic Testing for Avian Bornaviruses

Definitive diagnoses of the disease in living birds involve examination of histological lesions during biopsies or the use of radiology/ultrasonography to reveal the appearance of structures in the gastrointestinal tract [13]. Yet, bird owners tend to be concerned about the stress for their pets during such procedures, particularly if sedation is required. Furthermore, according to some researchers, only about 76% of birds with this disease have crop lesions and false-negative crop biopsy results tend to occur about 24% of the time [32]. Other researchers have reported a high level of variability between afflicted birds regarding locations of lesions in the gastrointestinal tract [33]. Thus, less invasive diagnostic testing tends to be preferred by both veterinarians and parrot owners, although low reliability and lack of standardization of laboratory tests remain an issue today [1].

### 7.1. Sample Collection

Diagnostic testing may involve swabbing the cloaca or collecting a bird's droppings, but these tests have limited usefulness due to variability among birds in how often the virus is shed through these routes. Some infected birds may consistently shed the bornaviruses, while others do so intermittently [4]. In fact, a single negative test in this context is typically not considered meaningful, so veterinarians and testing laboratories may recommend that samples be collected in a single container three times at weekly intervals [34]. Other testing laboratories may require other sample types such as the submission of whole blood and a cloacal swab to test for bornaviruses [35]. In their discussion of avian bornavirus diagnostics, Rossi et al. recommended that whole blood be sampled, in addition to choanal/cloacal swabs, but not fecal samples. Another potential diagnostic sampling technique involves testing the calami of plucked contour feathers [36]. The calamus of a feather is the tube at the base of a feather below the colored barbs. A contour feather is one that is located on the body of a bird, rather than the wings or tail. This type of sampling may be more sensitive because it is less likely to be affected by contaminants or by substances in blood and urofecal matter that can inhibit the test results. Furthermore, evidence suggests that feathers stored in baggies at room temperature for four weeks can remain useful for diagnostic testing. On the contrary, critics have pointed out that testing with feathers may lead to false positives due to contamination from other birds sharing the environment. Therefore, testing of plucked feathers may be more useful for detecting the presence of avian bornaviruses in aviaries or group-housed parrots rather than for individual birds [37]. However, plucking feathers is painful for birds, particularly when test laboratories require multiple feathers to be plucked (e.g., 4 to 8 feathers) [38]. In contrast, blood testing is a well-accepted veterinary procedure with parrots that theoretically at least allows for detection of the virus, detection of avian bornavirus antibodies, or genetic testing for subtypes of the avian bornaviruses. To conclude, veterinarians need to examine the sampling protocols of specific diagnostic laboratories which often vary in whether whole blood, serum, or plasma samples should be collected to conduct blood tests for avian bornaviruses. Hopefully, further research will reveal an optimum sampling protocol that can then be standardized across laboratories.

### 7.2. Diagnostic Assessments

The current recommended procedure for avian bornavirus testing is the reverse transcription polymerase chain reaction (RT-PCR) test, preferably in real time to monitor the progress of the PCR [1]. This is a two-step process involving reverse transcription to synthesize the genetic material and a polymerase chain reaction to amplify specific genetic targets [1, 37]. However, these tests typically only test for one or two specific subtypes of the virus, and there may, in fact, be other subtypes that remain to be discovered. At this time, there is no reliable ELISA (enzyme-linked immunosorbent assay) commercially available for detecting avian bornavirus antigens or antibodies from blood samples [39]. Researchers have pointed out that not all birds react strongly to the avian bornavirus antigen and therefore ELISA tests tend to lack specificity [37], and others have recommended a combination of RT-PCR and the western blot test to identify infected parrots [33], although the indirect fluorescent antibody (IFA) test tends to be used in Europe more often than the western blot test [1]. Both antibody tests, i.e., the western blot and the IFA test, are considered sensitive and specific, but they do not distinguish between diseased birds and healthy carrier birds [1]. Furthermore, there are discrepancies between viral shedding, the presence of antibodies, and clinical signs of the disease, with university and commercial laboratories ranging from 3 to 33% in positive avian bornavirus test results [1]. Instead, according to Rossi and his colleagues, serologic testing for antiganglioside antibodies may ultimately provide more accurate testing, given their reports that 98% of clinically symptomatic and histologically positive birds had elevated antiganglioside antibody levels in 650 avian serum samples [1]. It is possible that testing for antiganglioside antibodies may prove more reliable than directly testing for the virus or antibodies to the virus. Clearly, research in this context is ongoing and necessary.

## 8. Care and Treatment of Afflicted Parrots

Strong evidence suggests that birds with bornavirus infections can remain clinically healthy for decades [11], particularly if supportive care is provided. However, the identification of bornavirus-positive birds should be followed by containment, quarantining infected birds, and then conducting a thorough cleaning of infected aviaries. Fortunately, the avian bornavirus is not long-lived in the environment, and its spread can be limited by good hygiene and ultraviolet light. Research shows that the avian bornaviruses can be detected in the air of aviaries with infected birds. Researchers have described laboratory testing of air filters from dry-filter (DFR 1000) units which were consistently positive for the virus [31]. However, bornaviruses can be inactivated with chlorine-containing disinfectants [40], and research suggests that allowing infected cages to dry thoroughly before reuse is effective for removing avian bornaviruses. Specifically, in vitro testing revealed that 8 and 24 hours of drying reduced the amount of infectious virus by 48 and 86%, respectively [41]. Although they are believed to be stable at neutral pH and able to withstand both acidic and alkaline solutions, bornaviruses become rapidly less infective with heat treatment at 56 degrees Celsius (or 132.8 degrees Fahrenheit) and are relatively stable at 37 degrees Celsius (or 98.6 degrees Fahrenheit) [42]. *It is important to emphasize that parrots that test positive for these viruses should not be euthanized because they can live for years without clinical symptoms of the disease.* Daily care of birds with clinical symptoms of bornaviruses consists of providing an easily digestible, high energy diet, including banana, the mashed inside of potatoes or yams, rice, canned pumpkin, frozen peas, and scrambled eggs. On the contrary, seeds, nuts, strawberries, and the skin of fruits such as apples should be avoided because they are more difficult to digest. For this reason, some experts [13] also recommend semielemental diets designed to be highly nutritious and easily digestible for compromised birds [43]. Others have suggested that it may be beneficial to supplement the diets of birds in early stages of the disease with vegetables high in fiber because it may stimulate intestinal motility, while the same foods are undesirable for birds with more progressive symptoms because they may remain in the intestines and ferment [44]. While it is not yet clear what triggers the actual development of clinical signs in an infected bird, environmental stressors are likely to play a role and there is no doubt that a good diet is beneficial, as is some exposure to sunlight and good hygiene.

### 8.1. Currently Used Drug Therapies

Current veterinary treatments for birds that show clinical symptoms include anti-inflammatory drugs such as celecoxib, also known as Celebrex [45], a nonsteroidal pain-relieving anti-inflammatory drug, produced in a variety of flavors such as pumpkin or strawberry which can be attractive to parrots. Depending on how easily a bird can be handled, medication can be placed directly inside the bird's beak or added to its food or water. An alternative drug used to treat this disease is meloxicam [46]. Celebrex and meloxicam are both nonsteroidal anti-inflammatory drugs (NSAIDs) that inhibit cyclooxygenase (COX) isozymes, categorized as COX-1 (cyclooxygenase 1, i.e., the innate maintenance form of enzyme in the immune system that is present in most cells and tissues important for maintaining the integrity of the gastrointestinal mucosa, renal blood flow, and platelet aggregation) and COX-2 (cyclooxygenase 2) which is the inducible form of enzyme that is responsive to inflammatory stimuli and injury [47]. All NSAIDs are COX-2 inhibitors with some degree of COX-1 inhibition [48]. Celebrex is thought to be more selective to COX-2 enzymes than meloxicam and to have less effect on COX-1 processes [47]. Although Miesle stated that “…only one, Celebrex, has shown continual, long-term relief from gastrointestinal and Central Nervous System signs,” (p. 22) [7], the use of these anti-inflammatory drugs is still under debate and published citations of evidence about drug effectiveness are sometimes inaccurate and confusing [1, 3]. However, Dahlhausen et al. [49] published a brief description of eight cases of the disease in Watchbird, the Journal of the American Federation of Aviculture in which they described the results of treating afflicted parrots with Celebrex. They stated that, at therapeutic doses (10 mg/kg orally once daily), Celebrex does not affect COX-1 processes and that the drug was well tolerated and safe for the birds. They further stated that drug administration continued for 6 to 12 weeks until the birds returned to normal body weights, conditions, and diets. This occurred even for birds that were in advanced stages of the disease. If the birds were removed from the drug therapy and then showed the return of clinical signs, then they were successfully treated by restarting the drug. Dahlhausen et al. [49] further indicated that clinical conditions of these afflicted birds tended to improve within the first week of therapy with gradual resolution of clinical symptoms. No adverse side effects were reported for these birds either during or following treatment, and one of these parrots had completed therapy for 1 1/2 years. On the contrary, other veterinarians have recommended a daily use of 20 mg/kg oral doses for afflicted birds, although the dosage may be doubled for birds that are difficult to handle requiring the medicine to be added to their food [44]. These veterinarians also indicated that there were treatment failures as a result of batches of the drug that were not used quickly because the stability of Celebrex in water was unknown; therefore, they recommended that new stocks be prepared weekly and the drug be stored with refrigeration [44, 50]. Furthermore, they reported that these treatments resulted in side effects of gastrointestinal bleeding for some birds and the development of hypersensitivity to the drug in a hyacinth macaw, pointing out that the drug is counterindicated for birds with renal disease because most NSAIDs are eliminated through renal clearance [44, 50]. However, it remains unclear what the mortality rates would have been without drug treatment and what percentage of the birds would have improved under natural conditions. Definitively resolving these issues would require more systematic investigations, preferably with matched controls, which are difficult to conduct in veterinary practices.

On the contrary, the use of these anti-inflammatory drugs to treat afflicted parrots has been criticized, with one researcher suggesting that there is limited, if any, effectiveness of using Celebrex and meloxicam and also pointing out that there can be serious side effects such as gastrointestinal irritation and bleeding [47]. In fact, treatment of the disease with meloxicam in cockatiels was reported to cause disease symptoms to worsen in cockatiels [51]. Following systematic investigations of drug effectiveness, Escandon reported that the use of these anti-inflammatory drugs (i.e., both Celebrex and meloxicam) did not alter the progression of the disease, the severity of clinical symptoms, pathological changes in viral RNA distribution, or viral shedding in cockatiels after experimental inoculation with the avian bornaviruses [47]. She therefore concluded that there is “…no justification for continuing the practice of administering NSAIDs to birds with clinical PDD” (proventricular dilatation disease, p. 39) and stated that “…unless proven otherwise NSAID use in PDD cases should be discontinued immediately” (p. 40) [47]. However, an examination of some online veterinary services reveals that both drugs are currently recommended for treatment of the disease [52, 53]. Other veterinary websites indicate that long-term, albeit low dosage, drug treatments are recommended [52]. In contrast, Dahlhausen et al. recommended that afflicted birds be taken off these drugs when symptoms abate [49]. Therefore, if anti-inflammatory drugs are prescribed, veterinarians and parrot owners need to make decisions regarding the long-term use of these drugs or whether to merely administer these drugs during flare-ups of symptoms.

An additional immunosuppressant drug has recently been tested for its effectiveness in treating avian bornavirus disease. According to researchers, the use of cyclosporine, a drug used in humans to treat autoimmune diseases and to prevent organ rejection during transplants, resulted in a reduction of clinical symptoms in cockatiels, albeit with large amounts of virus remaining in the body [54]. Other researchers have investigated the effects of ribavirin, a drug that reduces RNA replication across a broad array of RNA viruses, and showed that it reduced, but did not eliminate, viral replication *in vitro* for both mammalian and avian cells, especially if combined with type 1 interferon known to help regulate the immune system [55]. In Japan, researchers have tested the effects of favipiravir (T-705), an antiviral drug known to be effective against viruses that cause human hemorrhagic fevers, again *in vitro* and observed that it reduced both mammalian and avian bornaviruses, although at relatively high doses [56]. It remains to be seen if these *in vitro* findings will transfer to *in vivo* testing without harm to the test animals. To conclude, additional systematic research is necessary to clarify optimal treatment protocols based on the specific symptoms and disease progression in individual birds.

### 8.2. Dietary Supplements

There appears to be less controversy regarding the use of gastrointestinal prokinetic agents to improve digestive processes and antibacterial and antifungal testing and therapy due to the disease-altered gastrointestinal microbiome, probiotics and prebiotics to help restore the normal intestinal environment, and omega fatty acids and herbal supplements such as milk thistle to reduce inflammation (see Dahlhausen and Orosz, 2015, for a detailed formulary) [13].

### 8.3. Vaccination

In an ideal world, treatments for avian bornavirus disease would help a bird's immune system resist the effects of the virus, as well as eliminate the virus from the body of the bird, and vaccines could be developed to prevent the onset of symptoms. The development of vaccines for avian bornaviruses is currently in its infancy with a few studies to date showing progress toward this goal. On the contrary, mammalian bornaviruses have been extensively studied, and research using laboratory rats has shown that mammalian bornaviral disease is related to T-cell activity with immunosuppressive drugs providing effective treatments, specifically with surviving rats having Type 2 immune responses, while the rats that died showed Type 1 immune responses [54]. Therefore, some researchers have speculated that vaccines for bornaviruses that are designed to affect the type of immune response may be more effective than vaccines that directly affect virus levels [54]. Hameed and colleagues (2018) provided some support for this approach by vaccinating cockatiels with a killed parrot virus plus recombinant PPaBV-4 nucleoprotein (N) in alum, thereby protecting these birds against a virulent bornavirus isolate (PPaBV-2), but without reduction of viral levels in the organs at necropsy [54]. They speculated that this would limit the commercial use of this vaccination protocol, although modifying the dose or immunization schedule could yield more desirable results.

On the contrary, Runge and colleagues (2016) described experiments with cockatiels and canaries using live viral vector vaccines carrying the N and P genes of avian bornaviruses and a heterologous prime-boost vaccination regime that yielded positive tests for antibodies reactive to the virus [57]. However, the progress of the disease was delayed in these birds, not prevented, following exposure to a high viral challenge dose of avian bornaviruses [57]. They pointed out that, without fuller knowledge regarding the transmission of avian bornaviruses, it is difficult to identify the appropriate routes and viral dosages to use when testing new vaccines [57]. These researchers speculated that naturally occurring infections of the disease may involve lower challenge dosages. They therefore conducted additional experiments using 10-fold lower dosages of challenge infections and second booster vaccinations which appeared to be successful in protecting these birds against viral infection with reduced clinical signs, fewer gross lesions, and only one bird showing mononuclear infiltrations while also lowering viral loads in these birds [57].

Clearly, while parrot owners around the world eagerly await a fully effective treatment for this disease, the research is ongoing. It may be that additional studies exploring modifications in existing vaccination protocols will lead to the successful prevention of the disease, or it may be that furthering our knowledge about the functioning of microscopic cellular structures such as gangliosides will play a role in future treatment successes. Perhaps, a deeper understanding of genetic coding and disease transmission will provide this much needed research breakthrough. Some experts have suggested that birds that are positive to the virus may have a natural immunity to the disease and therefore may be the best breeders for the avicultural community [44]. This assumes that clinical dormancy for this disease in parrots has high heritability which is actually unknown at this time. But, it may be useful to investigate genetic differences between healthy carriers and parrots that succumb to the disease. In humans, some individuals, particularly northern Europeans, have been discovered to have a natural immunity to HIV when they are homozygous carriers of a mutated gene that changes the size and position of the receptors which prevents HIV from entering their cells [58]. Therefore, HIV researchers are hopeful that investigations of disease-resistant individuals or populations, combined with further understanding of genomic coding, will contribute to the development of new strategies for developing HIV vaccinations [59]. Likewise, veterinarians and bird owners remain hopeful that new strategies for vaccine development will prove successful for treating avian bornaviral ganglioneuritis and ultimately other emerging viral diseases in the future.

## 9. The Importance of Bornavirus Research across Species

It is common knowledge among bird experts and veterinarians that the avian bornaviruses are not harmful to humans and that they do not grow in mammalian tissue in laboratories. In fact, researchers studying the evolution of bornaviruses have suggested that avian bornaviruses diverged from their ancestral version about 300 years ago [24]. Thus, one may conclude that this divergence in genetic material involved mutations that produced an avian bornavirus that is incompatible with human physiology. On the contrary, mammalian bornaviruses that infect a variety of species, including horses, sheep, cattle, rhesus monkeys, and rodents, can be transmitted between animals and humans. Recently, mammalian bornaviruses were shown to be associated with encephalitis and deaths in three people in Germany with variegated squirrels bites identified as the source of transmission [22]. Mammalian bornaviruses have also been under scrutiny for possible roles in the development of depression, bipolar disorder, and other psychiatric disorders because the virus appears to target the limbic system which plays a dominant role in our emotions. While some research has supported this view [60], other researchers have been critical, suggesting that investigations with better control conditions failed to reveal bornaviruses in psychiatric patients with mood disorders [9, 61]. Still other researchers have expressed concern about the possibility of mammalian bornaviruses being spread through human organ transplants, given the possibility of their association with psychiatric disorders and the recent cases of encephalitis transmitted by variegated squirrel bites [62]. Clearly, the bornaviruses remain an important focus for researchers worldwide who are seeking knowledge on and remedies for diseases in humans and other animals. Importantly, avian bornaviruses are prevalent in various ecological systems around the world, having been detected in a variety of species of birds other than parrots, including waterfowl, raptors, and passerines, therefore affecting numerous swans, geese, gulls, and wild ducks [2, 11]. Mammalian bornaviruses have also been found in a variety of other animals in their natural habitats, including rodents and snakes [63, 64]. Clearly, there are numerous natural reservoirs for the various types of bornaviruses.

Researchers continue to monitor occurrences of the various types of bornaviruses in the global natural environment, also seeking effective treatments for bornavirus-transmitted diseases. It seems likely that acquiring a fuller understanding of the prevalence, transmission, and treatment of emerging or reemerging diseases will be beneficial as we encounter new ones in the future. The ongoing research regarding avian and other bornaviruses has enormous importance for treating our afflicted pets, as well as for protecting humans from zoonotic diseases.
